# Insights Into Genetic Landscape of Large Granular Lymphocyte Leukemia

**DOI:** 10.3389/fonc.2020.00152

**Published:** 2020-02-18

**Authors:** Antonella Teramo, Gregorio Barilà, Giulia Calabretto, Cristina Vicenzetto, Vanessa Rebecca Gasparini, Gianpietro Semenzato, Renato Zambello

**Affiliations:** ^1^Hematology and Clinical Immunology Section, Department of Medicine (DIMED), Padova University School of Medicine, Padova, Italy; ^2^Veneto Institute of Molecular Medicine (VIMM), Padova, Italy

**Keywords:** large granular lymphocyte (LGL), T-LGL leukemia (T-LGLL), chronic lymphoproliferative disease of NK cells (CLPD-NK), STAT3, STAT5b, mutation

## Abstract

Large granular lymphocyte leukemia (LGLL) is a chronic proliferation of clonal cytotoxic lymphocytes, usually presenting with cytopenias and yet lacking a specific therapy. The disease is heterogeneous, including different subsets of patients distinguished by LGL immunophenotype (CD8+ Tαβ, CD4+ Tαβ, Tγδ, NK) and the clinical course of the disease (indolent/symptomatic/aggressive). Even if the etiology of LGLL remains elusive, evidence is accumulating on the genetic landscape driving and/or sustaining chronic LGL proliferations. The most common gain-of-function mutations identified in LGLL patients are on *STAT3* and *STAT5b* genes, which have been recently recognized as clonal markers and were included in the 2017 WHO classification of the disease. A significant correlation between *STAT3* mutations and symptomatic disease has been highlighted. At variance, *STAT5b* mutations could have a different clinical impact based on the immunophenotype of the mutated clone. In fact, they are regarded as the signature of an aggressive clinical course with a poor prognosis in CD8+ T-LGLL and aggressive NK cell leukemia, while they are devoid of negative prognostic significance in CD4+ T-LGLL and Tγδ LGLL. Knowing the specific distribution of *STAT* mutations helps identify the discrete mechanisms sustaining LGL proliferations in the corresponding disease subsets. Some patients equipped with wild type *STAT* genes are characterized by less frequent mutations in different genes, suggesting that other pathogenetic mechanisms are likely to be involved. In this review, we discuss how the LGLL mutational pattern allows a more precise and detailed tumor stratification, suggesting new parameters for better management of the disease and hopefully paving the way for a targeted clinical approach.

## Introduction

The 2017 world health organization (WHO) classification includes the large granular lymphocyte (LGL) leukemia in the category of cytotoxic T and NK cell leukemia and lymphoma. LGL leukemia is a lymphoproliferative disorder, sustained by clonal mature T or NK cells, that configures T-LGL leukemia (T-LGLL) or the chronic lymphoproliferative disease of NK cells (CLPD-NK), respectively (1). T-LGLL is the most frequent form (about 85% of cases), whereas, CLPD-NK is less represented (10% of cases) (2). A third group of rare (incidence 5%) diseases accounts for aggressive T-LGLL and aggressive NK cell leukemia (ANKL), characterized by very poor prognosis (2). T-LGLs usually express the TCR αβ+, CD4–, CD8+ phenotype and the disease is referred to CD8+ T-LGLL. The heterogeneity of the disease is emphasized by the presence, in 10–15% cases, of a disorder sustained by TCR αβ+, CD4+, CD8+/– LGLs, defining the CD4+ T-LGLL. Beyond the expansions of T cells bearing the TCR αβ+, a minority of cases originates from TCRγδ+ cells (Tγδ LGLL) (3). In addition, some patients are characterized by a bi-phenotypical variant, identified by a concomitant T/NK cell clone, or by a switch from T to NK phenotype or *vice versa* (4).

The disease is asymptomatic in nearly 30% of cases, with lymphocytosis representing the only observed hematological abnormality (5). However, during the disease course in 60% of cases therapy is needed, mostly for cytopenia-related manifestations, symptomatic patients showing clinical features often related to neutropenia (2). Currently, no specific treatment is available for LGL disorders and the current therapy is based on immunosuppressive drugs (i.e., Methotrexate, Cyclophosphamide or Cyclosporine A) giving unsatisfying responses (6, 7).

The etiopathogenesis of LGL leukemia has not been established. A viral or autologous antigen has been claimed to trigger the initial lymphocytosis whose survival over the time is then maintained by the upregulation of several cell activating pathways (8, 9). Among these, the Janus kinase/signal transducer and activator of transcription (JAK/STAT) signaling is central to direct the cell toward survival, being STAT an inducer of the transcription of many pro-survival genes (10). Supporting the role of these activatory pathways, in about 40% of patients, mutations on *STAT3* and *STAT5b* have been recognized as the most common gain-of-function genetic lesions up to now identified in LGLL patients. The resulting constitutive activation of STAT3 and STAT5b promotes an upregulation of the expression of genes that are required for cell proliferation and survival, i.e., *c-Myc, cyclin D1* and *cyclin D2, Bcl-xl, Mcl1*, and *survivin* (11). *STAT3* and *STAT5b* mutations have been included in the 2017 WHO LGLL classification (12).

## Genetics of T-LGLL

### *STAT3* Mutations

**Figure 1:**
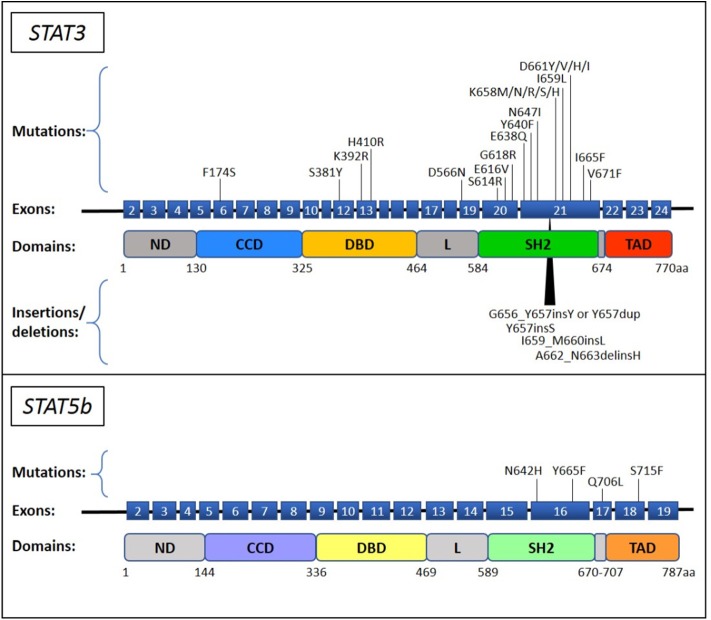
*STAT3* and *STAT5b* mutations described in T-LGLL and CLPD-NK. The *STAT3* and *STAT5b* mutations, reported up to now in literature (13–32), are indicated on their position in the exon upstream the corresponding protein domain. The point mutations and the insertions/deletions are reported above and below the schematic representation of the gene, respectively. ND, N-terminal domain; CCD, coiled-coil domain; DBD, DNA binding domain; L, linker; SH2, Src Homology 2; TAD, transactivation domain.

Currently, *STAT3* mutations are the most commonly recognized genetic lesions in T-LGLL. Somatic *STAT3* mutations are preferentially located in the Src homology 2 (SH2) domain of the gene, leading to an increase of the stability of STAT3 protein dimerization that results in an enhanced transcriptional activity of pro-survival proteins (13). *STAT3* mutation is preferentially found in CD8+ T-LGLL (14) and some TCRγδ LGLL cases (15), its incidence among the entire cohort of T-LGLL ranging from 11 up to 75% based on different reports (13–26). Y640F and D661Y are the most frequent *STAT3* genetic lesions, accounting for about 60% of the recognized mutations. The remnant other less frequent mutations include both point mutations and insertion or deletions and are mostly found in SH2 domain (13–26), although some missense substitutions were described in DNA-binding and coiled coil domains [(27); Figure 1]. All T-LGLL patients are characterized by STAT3 activation, that is the hallmark of every T-LGLL, but a higher amount of the phosphorylated protein has been observed in cases with *STAT3* mutations (13, 14, 17). Functional studies revealed that even if in different locations, most of the reported mutations lead to a higher protein transcriptional activity and cytokine responsiveness (13, 27). Nevertheless, deep transcriptional expression studies in T-LGLL did not find significant differences that distinguish patients with and without *STAT3* mutations, which showed similar overexpression of STAT3-responsive genes (13, 17, 28, 33). These findings suggest that in patients devoid of *STAT3* mutations, other mechanisms or lesions can be responsible of the activation of STAT3 pathway.

### *STAT5b* Mutations

Another member of STAT protein family has been reported to carry gain-of-function mutations, namely *STAT5b*. Initially discovered in only 2% of CD8+ T-LGLL, specifically found in the aggressive form of LGLL (28), *STAT5b* mutations were subsequently identified in 15–55% of CD4+ T-LGLL (14, 15, 29), and in 19% of TCRγδ LGLL (15). To date, genetic alterations discovered in *STAT5b* are all point mutations located in the SH2 and in the transactivation domain of the gene (Figure 1). The most recurrent mutations are N642H and Y665F, both deemed to increase the protein activity. At variance with STAT3, STAT5b activation has been observed only in cell samples carrying the mutated protein, whereas the wild type protein is unphosphorylated, similarly to healthy controls (28). Notably, functional studies and transcriptional analysis verified that N642H is able to induce a strong protein activation and to characterize patients with a peculiar gene expression distinguishing them from other patients who are not equipped with this mutation (including patients with *STAT* wild type, *STAT3* mutation and with *STAT5b* Y665F) (28).

### *TNFAIP3* Mutations

*TNF*α*-induced protein 3* (*TNFAIP3*) is another gene recurrently mutated in T-LGLL, that has been found altered in 4 cases within a cohort of 39 patients (24). This gene is a tumor suppressor that encodes A20, a negative regulator of nuclear factor kappa B (NF-kB), whose mutated form likely contributes to deregulate NF-kB activity. Moreover, the association between *TNFAIP3* and *STAT3* mutations (3 out of 4 cases simultaneously carry both mutations) suggests that *TNFAIP3* lesion is not only an alternative genetic mechanism to *STAT* alterations, but rather an additional event concurring to induce the LGLL phenotype.

### Other Less Frequent Gene Mutations

Through deep sequencing, some authors discovered other genes occasionally mutated in T-LGLL, many of them linked to STAT3 signaling pathway and cytotoxic T lymphocyte activation, including *PTPRT, BCL11B, PTPN14, PTPN23* (13, 27–29, 33–35). Consistently, a systems genetic assay showed that 5 out of 8 patients devoid of *STAT* mutations carried alterations on genes “STAT related” or connecting STAT with Ras/MAPK/ERK and IL-15 signaling, including *FLT3, ANGPT2, KDR/VEGFR2*, and *CD40LG* (36). These data emphasize the central role of JAK/STAT pathway in T-LGLL patients regardless of *STAT* mutational pattern. In addition, this analysis demonstrates that several genetic lesions affect different functionally connected genes that can concur to drive a similar phenotype.

## Genetics of CLPD-NK

### *STAT3* and *STAT5b* Mutations

Although sharing many similarities (37), CLPD-NK is only partially reminiscent of the genetic background of T-LGLL. Since this disease is rarer than the T related entity, less data are available and appropriate analyses are lacking to precisely define this disorder. The similarity with T-LGLL involves *STAT3* gene, that is reported mutated also in CLPD-NK [(17, 38); Figure 1]. To note, our data indicate that *STAT3* mutations seem to have a lower incidence, from 5.9 to 8.3%, in this disease as compared to T-LGLL (15, 39). Other studies report a frequency of *STAT3* mutations from 11 up to 40%; the two studies with the largest number of CLPD-NK patients indicate 15/50 (30%) and 5/40 (13%) cases with *STAT3* mutation (17, 40).

Differently from T-LGLL, CLPD-NK appears to be devoid of *STAT5b* genetic lesions (15, 38, 39), with the only exception of the aggressive case discussed by Rajala et al., who subsequently developed ANKL (28).

### Other Less Frequent Gene Mutations

In line with T-LGLL, *TNFAIP3* mutation has been found in one out of 17 CLPD-NK patients (5.9%) (38).

Other genetic alterations have been detected through whole exome sequencing (WES) on 3 CLPD-NK patients (all *STAT*-mutation-negative) by Coppe et al. (36). From this analysis, 31 genes harbored somatic mutations including several “cancer genes” i.e., *KRAS, PTK2, NOTCH2, CDC25B, HRASLS, RAB12, PTPRT*, and *LRBA*. More recently, the same authors reported WES data obtained in a larger series of patients indicating that the involvement of JAK/STAT pathway resulted to be not so central as observed in T-LGLL. Otherwise, other pathways and genes were hit by genetic alterations potentially impacting on cell survival, proliferation, chromatin-remodeling, innate immunity, and NK cells activation (41).

Currently, more information is available on ANKL rather than CLPD-NK. Through WES on 14 ANKL patients, Dufva et al. identified alterations in JAK/STAT, RAS/MAPK, and epigenetic modifier genes (42). They found JAK/STAT signaling components frequently altered, with 21% of cases carrying *STAT3* mutations, showing that some features are shared by ANKL and the relatively indolent LGLL. The same authors demonstrated an overlapping genetic landscape between ANKL and extranodal NK/T cell lymphoma, nasal type (NKTCL) rather than with CLPD-NK, suggesting that ANKL might represent a more advanced form of NKTCL (42).

## *STAT* Mutation: Founding or Late Event?

The current pathogenetic hypothesis on LGLL development rests on an initial antigen-triggered oligoclonal LGL expansion that only successively develops into a monoclonal lymphocytosis. In this context, *STAT* mutation seems not to be an inciting but rather an acquired event during the disease, conferring an advantage on the clone development. Indeed, *STAT* mutations are more frequently found in large clones (18). Interestingly, *STAT5b* mutations are more often reported on large monoclonal TCR-Vβ expansions (28), whereas, *STAT3* mutations are also detected in small subclones (18). Kerr et al., evaluating the relationship between *STAT3* mutation and T-cell clone burden, showed that *STAT3* mutation frequency can be lower than the T-cell clone entity thus confirming that the mutation is likely to occur as a secondary event within a pre-expanded immunodominant clone (43). The above authors also observed that *STAT3* mutation may contribute to an autonomous antigen-independent clonal expansion (43).

Many data have been reported demonstrating that *STAT3* mutation does not represent the only factor, itself mandatory, to trigger LGL clonal expansion. *In vitro* inhibition of STAT3 was observed to restore LGL apoptosis independently from *STAT3* mutational status and STAT3 was found activated also in *STAT3* wild type LGLL patients (17). Furthermore, analysis on murine cells transduced with retrovirus showed that *STAT3* mutants (D661V, Y640F) do not provide any cell growth advantage (44). Similarly, a mouse model demonstrated that the expression of *STAT3* mutant alone is not enough to induce LGL leukemia (44), at variance to what had been observed in the mouse model with over-production of IL15 (45). In addition, in a murine bone marrow transplantation model, also Couronne et al. showed that the expression of Y640F mutated STAT3 primarily induces myeloid malignancy rather than LGL disease (46). All these results suggest that additional gene mutations or deregulation due to other signaling molecules or pathways associated with *STAT3* mutations might be involved in LGLL pathogenesis (47).

Several points should be made in terms of the *STAT5b* mutations. *STAT5b* N642H has been indeed identified as an oncogenic driver in innate-like lymphocytes (48), and a mouse model expressing human N642H mutated STAT5b has been described to develop severe CD8+ T cell neoplasia (49). Provided that IL-15 is an upstream factor of STAT5b and that IL-15 transgenic mice develops the aggressive variant of T or NK cell leukemia (50), the IL-15-STAT5 axis might be considered crucial for neoplastic transformation. The requirement of additional cytokine signals on *STAT5b* genetic lesions suggests that the indolent course of CD4+ T-LGLL or Tγδ LGLL carrying these genetic lesions might be due to the lack of one or more concurring events together with *STAT5b* mutations, e.g., cytokine stimulation.

## The Clinical Impact of *STAT3* and *STAT5b* Mutations

Isolated neutropenia represents the clinical hallmark of the disease, observed in 40–60% of patients, with approximately half of them developing severe neutropenia. A significant correlation between the presence of *STAT3* mutations and neutropenia/symptomatic disease has already been highlighted in several studies (13–16, 22, 25). We hypothesized that the mechanism accounting for neutropenia development involves high levels of STAT3 activation (14). More in detail, we recently demonstrated the presence of a STAT3-miR-146b-FasL axis in neutropenic T-LGLL patients, that, once triggered, leads to high production of Fas Ligand, which in turn is responsible of neutrophil apoptosis (51). These data emphasize the role of STAT3 activation in the pathogenesis of LGLL neutropenia, with *STAT3* mutations likely being involved in further boosting this mechanism.

Besides neutropenia, several other clinical features have been described to be more frequent in patients with *STAT3* mutations, including different cytopenias or autoimmune diseases. Interestingly, T-LGLL patients with multiple *STAT3* mutations have been reported to associate with concomitant rheumatoid arthritis (RA) (52). On the contrary, the association with pure red cell aplasia (PRCA) remains a controversial issue (17, 21, 22, 26).

At variance with *STAT3, STAT5b* mutation has been reported to have a very different clinical impact. Depending on the immunophenotype of the mutated clone, the presence of *STAT5b* mutations in the same hotspot positions represents a signature of aggressive clinical course with a poor prognosis in aggressive CD8+ T-LGLL patients (28), while it is devoid of negative prognostic significance in CD4+ T-LGLL and Tγδ LGLL patients (15, 29, 53). The issue is quite intriguing since *STAT5b* N642H behaves as a driver mutation in several T-cell lymphomas and in the mice model it is enough to induce a leukemic phenotype (48).

The association between *STATs* mutations and patients' clinical features has been recently confirmed by our data obtained in a large cohort of 205 LGLL patients including all LGLL subtypes, but aggressive T-LGLL and ANKL. We observed that *STAT3* mutations were significantly associated with absolute neutrophils count <500/mm^3^, hemoglobin level <90 g/L and treatment requirement, while *STAT5b* mutations were found in 15/152 asymptomatic patients. Moreover, by univariate and multivariate analysis, *STAT3* mutated status resulted to be associated with reduced overall survival, firstly demonstrating the adverse impact of *STAT3* mutations in LGLL patients (15).

Considering that a specific therapy is still missing in LGLL and that current immunosuppressive drugs do not provide satisfying responses, the above-mentioned clinical impact of STAT signaling in LGLL makes these molecules attractive new targets for drug development. Several direct STAT inhibitors interacting with protein domains are available, including Stattic, S3I-201, STA-21 for STAT3 (54) and Pimozide, Stafib2, and Cpd17f for STAT5b (55). However, these compounds induce several off-targets toxicities and severe side-effects that for the time being prevent their use in the clinical setting. To date, no direct STAT3/5b inhibitors of clinical grade are available. However, early-phase clinical trials with drugs targeting STAT3 are ongoing in solid and hematologic malignancies other than LGLL, namely AZD9150, a STAT3 antisense oligonucleotide, and Napabucasin, an inhibitor of gene expression driven by STAT3 (56). In LGLL some compounds against the upstream signaling to STAT have been tested, with preliminary promising results. Tofacitinib citrate, a JAK3-specific inhibitor, showed good response in patients with refractory LGLL associated with RA (30); furthermore, BNZ-1, a multicytokine inhibitor, is currently being tested in LGLL patients in a phase I/II trial (57). Additional studies are needed to confirm these data and the inclusion of *STAT* mutational status in the work-up is suggested to achieve a personalized treatment of LGLL. Consistently, *STAT3* Y640F mutations have been shown to predict response to methotrexate in a small series of patients (7), representing a putative, potential parameter to select the initial best therapy for LGLL patients.

## *STAT3* and *STAT5b* Mutations Occur in Phenotypically Distinct LGLL

The correlation found between *STAT* mutations and LGL immunophenotype has been highlighted, in fact *STAT3* and *STAT5b* mutations are mutually exclusive and preferentially occur in phenotypically distinct leukemic LGLs.

Within Tαβ-LGL disorders, *STAT3* mutations characterize the CD8+ T-LGLL and have never been observed in the CD4+ subset (14, 29). Instead, *STAT5b* mutations have been mainly found in CD4+ TLGLL and also in the rare aggressive form of CD8+ T-LGLL (28), whereas in indolent CD8+ T-LGLL these genetic lesions seem to be rare (14, 29).

Besides this preliminary distinction, a more precise definition of patients harboring *STAT* mutation can be described, considering the differential immunophenotypic combination of the LGL markers, i.e., CD16, CD56, and CD57. In CD8+ T-LGLL, the CD16+/CD56– phenotype, with or without CD57, is strongly linked to patients characterized by the presence of *STAT3* mutation (14). The rare aggressive form of T-LGLL, frequently carrying *STAT5b* mutations, is discretely characterized by the proliferation of CD8+/CD56+/CD16–/CD57– LGLs (28). Interestingly, *STAT5b* mutation is found among CD4+ TLGLL patients whose LGL clone is always CD56+, CD16– (14, 29). Similarly, even though CD16 frequently characterizes Tγδ LGLs, in Tγδ LGLL we observed *STAT3* mutations in CD56– LGLs, whereas *STAT5b* mutations were detected in CD56+ phenotype (53).

Also in CLPD-NK discrete subtypes can be identified by flow analysis. We observed that patients with CD56^−/dim^/CD16^high^/CD57^−^ cytotoxic NK cells expansion include a subgroup characterized by a more symptomatic disease and the presence of *STAT3* mutation (39). For ANKL, otherwise, no *STAT* mutation-linked phenotypes have been reported.

**Figure 2:**
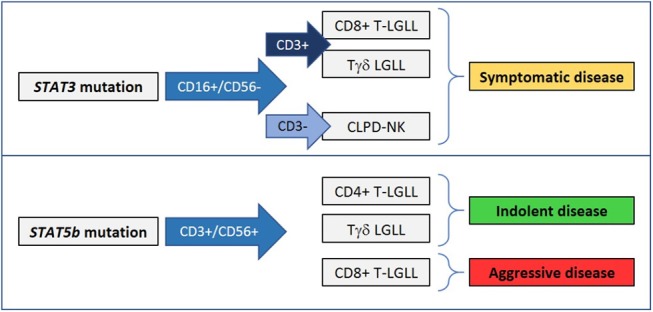
*STAT3* and *STAT5b* mutations are preferentially found in phenotypically distinct LGL disorders and can correlate with different clinical presentations.

Taken together, these data suggest that *STAT3* mutation can occur in CD16+/CD56– LGLs, whereas *STAT5b* mutation may be detectable in CD56+ LGL (Figure 2). This link suggests that CD16+/CD56– LGLs and CD56+ LGLs preferentially use *STAT3* or *STAT5b* signaling, respectively, to develop LGL expansion and consequently they are differentially predisposed to be genetically hit. The nature of this relationship remains an open issue that needs to be elucidated. A larger analysis on CLPD-NK and Tγδ LGLL cases is mandatory to get insights also in these less frequent disorders.

## Conclusions

The gain-of-function mutations in *STAT3* and *STAT5b* genes up to now remain the most frequent abnormalities in LGLL, even if additional genetic lesions in STAT-related or cancer genes have been described. The complexity on the genetic background indicates that LGLL can be the result of different mechanisms, emphasizing the heterogeneity of the disease, even if a highly frequent recurrent genetic event has not been demonstrated. Being the most common and specific for this disorder, *STAT3* mutation currently remains the genetic marker suggestive of LGLL, whereas *STAT5b* gene is frequently described mutated also in many other hematologic diseases beyond LGLL.

Molecular analysis of *STAT3* and *STAT5b* is unfortunately not so widely available nowadays, but the evidence that *STAT* mutations have a clinical impact supports the inclusion of this test in the LGLL diagnostic work-up. Moreover, the evaluation of *STAT* mutations is suggested in combination with LGL immunophenotype data to perform an appropriate classification of indolent, symptomatic and aggressive LGLL patients. *STAT3* mutation is indicative of symptomatic disease and reduced patient survival; its evaluation is preferentially suggested for patients affected by CD8 T-LGLL, Tγδ LGLL, and CLPD-NK, particularly when clonal expansion is sustained by CD16+/CD56– LGLs. *STAT5b* mutation has a controversial significance depending on the immunophenotype of the mutated clone. In fact, it is regarded as the signature of an aggressive clinical course with a poor prognosis in CD8+ TLGLL, characterized by CD56+/CD16–/CD57– LGLs, and aggressive NK cell leukemia, while it is devoid of negative prognostic significance in CD4+ T-LGLL and Tγδ LGLL. Unfortunately, the key factors addressing these two different clinical courses are currently unknown.

*STAT3* and *STAT5b* mutations have been included in the 2017 WHO classification of LGLL with the indication that *STAT5b* mutation is associated with a more aggressive disease (12). Now this statement needs to be updated (i) highlighting the correlation between *STAT3* mutation, symptomatic disease and short patient survival and (ii) adding the issue of discovery of *STAT5b* genetic lesions also to indolent CD4+ T-LGLL and Tγδ LGLL. Moreover, in terms of aggressive LGLL harboring *STAT5b* mutation, the current WHO classification recognizes only ANKL, but does not yet recognize the T-related variants as a separate entity. Considering all these findings, the introduction of *STAT* mutation screening as diagnostic tool, together with a correct immunophenotypic analysis, is encouraged for an accurate characterization of LGLL patients.

## Author Contributions

AT wrote the manuscript. AT, GB, and GC discussed the topics of the manuscript. CV and VG contributed to check the data contained in the manuscript. GS and RZ reviewed, edited, and approved the final version of the manuscript.

### Conflict of Interest

The authors declare that the research was conducted in the absence of any commercial or financial relationships that could be construed as a potential conflict of interest.

## References

[1] Zambello R Semenzato G. Large granular lymphocyte disorders: new etiopathogenetic clues as a rationale for innovative therapeutic approaches. Haematologica. (2009) 94:1341–5. 10.3324/haematol.2009.012161 19794080 PMC2754948

[2] Lamy T Moignet A Loughran TP Jr. LGL leukemia: from pathogenesis to treatment. Blood. (2017) 129:1082–94. 10.1182/blood-2016-08-692590 28115367

[3] Barila G Calabretto G Teramo A Vicenzetto C Gasparini VR Semenzato G. T cell large granular lymphocyte leukemia and chronic NK lymphocytosis. Best Pract Res Clin Haematol. (2019) 32:207–16. 10.1016/j.beha.2019.06.006 31585621

[4] Gattazzo C Teramo A Passeri F De March E Carraro S Trimarco V. Detection of monoclonal T populations in patients with KIR-restricted chronic lymphoproliferative disorder of NK cells. Haematologica. (2014) 99:1826–33. 10.3324/haematol.2014.105726 25193965 PMC4258759

[5] Semenzato G Pandolfi F Chisesi T De Rossi G Pizzolo G Zambello R. The lymphoproliferative disease of granular lymphocytes. A heterogeneous disorder ranging from indolent to aggressive conditions. Cancer. (1987) 60:2971–8. 10.1002/1097-0142(19871215)60:12<2971::AID-CNCR2820601220>3.0.CO;2-O 3677021

[6] Moignet A Lamy T. Latest advances in the diagnosis and treatment of large granular lymphocytic leukemia. Am Soc Clin Oncol Educ Book. (2018) 38:616–25. 10.1200/EDBK_200689 30231346

[7] Loughran TP Jr Zickl L Olson TL Wang V Zhang D Rajala HL. Immunosuppressive therapy of LGL leukemia: prospective multicenter phase II study by the Eastern Cooperative Oncology Group (E5998). Leukemia. (2015) 29:886–94. 10.1038/leu.2014.298 25306898 PMC4377298

[8] Zhang R Shah MV Yang J Nyland SB Liu X Yun JK. Network model of survival signaling in large granular lymphocyte leukemia. Proc Natl Acad Sci USA. (2008) 105:16308–13. 10.1073/pnas.0806447105 18852469 PMC2571012

[9] Zambello R Teramo A Barila G Gattazzo C Semenzato G. Activating KIRs in chronic lymphoproliferative disorder of NK cells: protection from viruses and disease induction? Front Immunol. (2014) 5:72. 10.3389/fimmu.2014.00072 24616720 PMC3935213

[10] Epling-Burnette PK Liu JH Catlett-Falcone R Turkson J Oshiro M Kothapalli R. Inhibition of STAT3 signaling leads to apoptosis of leukemic large granular lymphocytes and decreased Mcl-1 expression. J Clin Invest. (2001) 107:351–62. 10.1172/JCI9940 11160159 PMC199188

[11] Yu H Jove R. The STATs of cancer–new molecular targets come of age. Nat Rev Cancer. (2004) 4:97–105. 10.1038/nrc1275 14964307

[12] Matutes E. The 2017 WHO update on mature T- and natural killer (NK) cell neoplasms. Int J Lab Hematol. (2018) 40(Suppl. 1):97–103. 10.1111/ijlh.12817 29741263

[13] Koskela HL Eldfors S Ellonen P van Adrichem AJ Kuusanmaki H Andersson EI. Somatic STAT3 mutations in large granular lymphocytic leukemia. N Engl J Med. (2012) 366:1905–13. 10.1056/NEJMoa1114885 22591296 PMC3693860

[14] Teramo A Barila G Calabretto G Ercolin C Lamy T Moignet A. STAT3 mutation impacts biological and clinical features of T-LGL leukemia. Oncotarget. (2017) 8:61876–89. 10.18632/oncotarget.18711 28977911 PMC5617471

[15] Barila G Teramo A Calabretto G Vicenzetto C Gasparini VR Pavan L. Stat3 mutations impact on overall survival in large granular lymphocyte leukemia: a single-center experience of 205 patients. Leukemia. (2019). 10.1038/s41375-019-0644-0. [Epub ahead of print]. 31740810

[16] Ohgami RS Ma L Merker JD Martinez B Zehnder JL Arber DA. STAT3 mutations are frequent in CD30+ T-cell lymphomas and T-cell large granular lymphocytic leukemia. Leukemia. (2013) 27:2244–7. 10.1038/leu.2013.104 23563237

[17] Jerez A Clemente MJ Makishima H Koskela H Leblanc F Peng Ng K. STAT3 mutations unify the pathogenesis of chronic lymphoproliferative disorders of NK cells and T-cell large granular lymphocyte leukemia. Blood. (2012) 120:3048–57. 10.1182/blood-2012-06-435297 22859607 PMC3471515

[18] Fasan A Kern W Grossmann V Haferlach C Haferlach T Schnittger S. STAT3 mutations are highly specific for large granular lymphocytic leukemia. Leukemia. (2013) 27:1598–600. 10.1038/leu.2012.350 23207521

[19] Kristensen T Larsen M Rewes A Frederiksen H Thomassen M Moller MB. Clinical relevance of sensitive and quantitative STAT3 mutation analysis using next-generation sequencing in T-cell large granular lymphocytic leukemia. J Mol Diagn. (2014) 16:382–92. 10.1016/j.jmoldx.2014.02.005 24797340

[20] Clemente MJ Przychodzen B Jerez A Dienes BE Afable MG Husseinzadeh H. Deep sequencing of the T-cell receptor repertoire in CD8+ T-large granular lymphocyte leukemia identifies signature landscapes. Blood. (2013) 122:4077–85. 10.1182/blood-2013-05-506386 24149287 PMC3862272

[21] Ishida F Matsuda K Sekiguchi N Makishima H Taira C Momose K. STAT3 gene mutations and their association with pure red cell aplasia in large granular lymphocyte leukemia. Cancer Sci. (2014) 105:342–6. 10.1111/cas.12341 24350896 PMC4317942

[22] Qiu ZY Fan L Wang L Qiao C Wu YJ Zhou JF. STAT3 mutations are frequent in Tcell large granular lymphocytic leukemia with pure red cell aplasia. J Hematol Oncol. (2013) 6:82. 10.1186/1756-8722-6-82 24283217 PMC4222121

[23] Rajala HL Olson T Clemente MJ Lagstrom S Ellonen P Lundan T. The analysis of clonal diversity and therapy responses using STAT3 mutations as a molecular marker in large granular lymphocytic leukemia. Haematologica. (2015) 100:91–9. 10.3324/haematol.2014.113142 25281507 PMC4281318

[24] Johansson P Bergmann A Rahmann S Wohlers I Scholtysik R Przekopowitz M. Recurrent alterations of TNFAIP3 (A20) in T-cell large granular lymphocytic leukemia. Int J Cancer. (2016) 138:121–4. 10.1002/ijc.29697 26199174

[25] Sanikommu SR Clemente MJ Chomczynski P Afable MG II Jerez A Thota S. Clinical features and treatment outcomes in large granular lymphocytic leukemia (LGLL). Leuk Lymphoma. (2018) 59:416–22. 10.1080/10428194.2017.1339880 28633612 PMC8694069

[26] Shi M He R Feldman AL Viswanatha DS Jevremovic D Chen D. STAT3 mutation and its clinical and histopathologic correlation in T-cell large granular lymphocytic leukemia. Hum Pathol. (2018) 73:74–81. 10.1016/j.humpath.2017.12.014 29288042

[27] Andersson E Kuusanmaki H Bortoluzzi S Lagstrom S Parsons A Rajala H. Activating somatic mutations outside the SH2-domain of STAT3 in LGL leukemia. Leukemia. (2016) 30:1204–8. 10.1038/leu.2015.263 26419508 PMC4814354

[28] Rajala HL Eldfors S Kuusanmaki H van Adrichem AJ Olson T Lagstrom S. Discovery of somatic STAT5b mutations in large granular lymphocytic leukemia. Blood. (2013) 121:4541–50. 10.1182/blood-2012-12-474577 23596048 PMC3668487

[29] Andersson EI Tanahashi T Sekiguchi N Gasparini VR Bortoluzzi S Kawakami T. High incidence of activating STAT5B mutations in CD4-positive T-cell large granular lymphocyte leukemia. Blood. (2016) 128:2465–8. 10.1182/blood-2016-06-724856 27697773 PMC5114490

[30] Bilori B Thota S Clemente MJ Patel B Jerez A Afable Ii M. Tofacitinib as a novel salvage therapy for refractory T-cell large granular lymphocytic leukemia. Leukemia. (2015) 29:24279. 10.1038/leu.2015.280 26449659

[31] Haapaniemi EM Kaustio M Rajala HL van Adrichem AJ Kainulainen L Glumoff V. Autoimmunity, hypogammaglobulinemia, lymphoproliferation, and mycobacterial disease in patients with activating mutations in STAT3. Blood. (2015) 125:639–48. 10.1182/blood-2014-04-570101 25349174 PMC4304109

[32] Morgan EA Lee MN DeAngelo DJ Steensma DP Stone RM Kuo FC. Systematic STAT3 sequencing in patients with unexplained cytopenias identifies unsuspected large granular lymphocytic leukemia. Blood Adv. (2017) 1:1786–9. 10.1182/bloodadvances.2017011197 29296824 PMC5728102

[33] Andersson EI Rajala HL Eldfors S Ellonen P Olson T Jerez A. Novel somatic mutations in large granular lymphocytic leukemia affecting the STAT-pathway and T-cell activation. Blood Cancer J. (2013) 3:e168. 10.1038/bcj.2013.65 24317090 PMC3877422

[34] Andersson EI Coppe A Bortoluzzi S. A guilt-by-association mutation network in LGL leukemia. Oncotarget. (2017) 8:93299–300. 10.18632/oncotarget.21699 29212142 PMC5706788

[35] Raess PW Cascio MJ Fan G Press R Druker BJ Brewer D. Concurrent STAT3, DNMT3A, and TET2 mutations in T-LGL leukemia with molecularly distinct clonal hematopoiesis of indeterminate potential. Am J Hematol. (2017) 92:E6–8. 10.1002/ajh.24586 27761930

[36] Coppe A Andersson EI Binatti A Gasparini VR Bortoluzzi S Clemente M. Genomic landscape characterization of large granular lymphocyte leukemia with a systems genetics approach. Leukemia. (2017) 31:1243–6. 10.1038/leu.2017.49 28167832 PMC5419584

[37] Zambello R Teramo A Gattazzo C Semenzato G. Are T-LGL leukemia and NK-chronic lymphoproliferative disorder really two distinct diseases? Transl Med UniSa. (2014) 8:4–11. 24778993 PMC4000458

[38] Kawakami T Sekiguchi N Kobayashi J Yamane T Nishina S Sakai H. STAT3 mutations in natural killer cells are associated with cytopenia in patients with chronic lymphoproliferative disorder of natural killer cells. Int J Hematol. (2019) 109:563–71. 10.1007/s12185-019-02625-x 30859397

[39] Barila G Teramo A Calabretto G Ercolin C Boscaro E Trimarco V. Dominant cytotoxic NK cell subset within CLPD-NK patients identifies a more aggressive NK cell proliferation. Blood Cancer J. (2018) 8:51. 10.1038/s41408-018-0088-1 29891951 PMC6002482

[40] Poullot E Zambello R Leblanc F Bareau B De March E Roussel M. Chronic natural killer lymphoproliferative disorders: characteristics of an international cohort of 70 patients. Ann Oncol. (2014) 25:2030–5. 10.1093/annonc/mdu369 25096606 PMC4176455

[41] Gasparini VR Binatti A Teramo A Coppe A Vicenzetto C Calabretto G A first highdefinition landscape of somatic mutations in chronic lymphoproliferative disorder of NK cells. In: Jan Cools AE, editor. 24th Congress of the European Hematology Association. Amsterdam: HemaSphere (2019). p. 132–3. 10.1097/01.HS9.0000559660.30415.c9

[42] Dufva O Kankainen M Kelkka T Sekiguchi N Awad SA Eldfors S. Aggressive natural killer-cell leukemia mutational landscape and drug profiling highlight JAK-STAT signaling as therapeutic target. Nat Commun. (2018) 9:1567. 10.1038/s41467-018-03987-2 29674644 PMC5908809

[43] Kerr CM Clemente MJ Chomczynski PW Przychodzen B Nagata Y Adema V. Subclonal STAT3 mutations solidify clonal dominance. Blood Adv. (2019) 3:917–21. 10.1182/bloodadvances.2018027862 30898763 PMC6436009

[44] Dutta A Yan D Hutchison RE Mohi G. STAT3 mutations are not sufficient to induce large granular lymphocytic leukaemia in mice. Br J Haematol. (2018) 180:911–5. 10.1111/bjh.14487 28025836 PMC5484756

[45] Mishra A Liu S Sams GH Curphey DP Santhanam R Rush LJ. Aberrant overexpression of IL-15 initiates large granular lymphocyte leukemia through chromosomal instability and DNA hypermethylation. Cancer Cell. (2012) 22:645–55. 10.1016/j.ccr.2012.09.009 23153537 PMC3627362

[46] Couronne L Scourzic L Pilati C Della Valle V Duffourd Y Solary E. STAT3 mutations identified in human hematologic neoplasms induce myeloid malignancies in a mouse bone marrow transplantation model. Haematologica. (2013) 98:1748–52. 10.3324/haematol.2013.085068 23872306 PMC3815176

[47] Teramo A Gattazzo C Passeri F Lico A Tasca G Cabrelle A. Intrinsic and extrinsic mechanisms contribute to maintain the JAK/STAT pathway aberrantly activated in T-type large granular lymphocyte leukemia. Blood. (2013) 121:3843–54, S1. 10.1182/blood-2012-07-441378 23515927

[48] Klein K Witalisz-Siepracka A Maurer B Prinz D Heller G Leidenfrost N. STAT5B^N642H^ drives transformation of NKT cells: a novel mouse model for CD56^+^ T-LGL leukemia. Leukemia. (2019) 33:2336–40. 10.1038/s41375-019-0471-3 30967617 PMC6756040

[49] Pham HTT Maurer B Prchal-Murphy M Grausenburger R Grundschober E Javaheri T. STAT5BN642H is a driver mutation for T cell neoplasia. J Clin Invest. (2018) 128:387–401. 10.1172/JCI94509 29200404 PMC5749501

[50] Fehniger TA Suzuki K Ponnappan A VanDeusen JB Cooper MA Florea SM. Fatal leukemia in interleukin 15 transgenic mice follows early expansions in natural killer and memory phenotype CD8+ T cells. J Exp Med. (2001) 193:219–31. 10.1084/jem.193.2.219 11208862 PMC2193336

[51] Mariotti B Calabretto G Rossato M Teramo A Castellucci M Barila G. Identification of a miR-146b-FasL axis in the development of neutropenia in T large granular lymphocyte leukemia. Haematologica. (2019). 10.3324/haematol.2019.225060. [Epub ahead of print]. 31467122 PMC7193483

[52] Savola P Kelkka T Rajala HL Kuuliala A Kuuliala K Eldfors S. Somatic mutations in clonally expanded cytotoxic T lymphocytes in patients with newly diagnosed rheumatoid arthritis. Nat Commun. (2017) 8:15869. 10.1038/ncomms15869 28635960 PMC5482061

[53] Teramo A Ciabatti E Tarrini G Petrini I Barila G Calabretto G Clonotype and mutational pattern in TCRγδ large granular lymphocyte leukemia. In: Malcovati L, editor. 22nd Congress of the European Hematology Association. Madrid: Haematologica (2017). p. 573.

[54] Zhu F Wang KB Rui L. STAT3 activation and oncogenesis in lymphoma. Cancers. (2019) 12:E19. 10.3390/cancers12010019 31861597 PMC7016717

[55] Orlova A Wagner C de Araujo ED Bajusz D Neubauer HA Herling M. Direct targeting options for STAT3 and STAT5 in cancer. Cancers. (2019) 11:E1930. 10.3390/cancers11121930 31817042 PMC6966570

[56] Yang L Lin S Xu L Lin J Zhao C Huang X. Novel activators and small-molecule inhibitors of STAT3 in cancer. Cytokine Growth Factor Rev. (2019) 49:10–22. 10.1016/j.cytogfr.2019.10.005 31677966

[57] Frohna PA Ratnayake A Doerr N Basheer A Al-Mawsawi LQ Kim WJ. Results from a first-in-human study of BNZ-1, a selective multicytokine inhibitor targeting members of the common gamma (γc) family of cytokines. J Clin Pharmacol. (2019) 60:264–73. 10.1002/jcph.1522 31465127 PMC8317201

